# Robust B Cell Responses Predict Rapid Resolution of Lyme Disease

**DOI:** 10.3389/fimmu.2018.01634

**Published:** 2018-07-18

**Authors:** Lisa K. Blum, Julia Z. Adamska, Dale S. Martin, Alison W. Rebman, Serra E. Elliott, Richard R. L. Cao, Monica E. Embers, John N. Aucott, Mark J. Soloski, William H. Robinson

**Affiliations:** ^1^Stanford University School of Medicine, Stanford, CA, United States; ^2^VA Palo Alto Healthcare System, Palo Alto, CA, United States; ^3^Division of Bacteriology and Parasitology, Tulane National Primate Research Center, Tulane University Health Sciences Center, Covington, LA, United States; ^4^Lyme Disease Research Center, Division of Rheumatology, Department of Medicine, Johns Hopkins University School of Medicine, Baltimore, MD, United States

**Keywords:** Lyme disease, *Borrelia*, antibodies, plasmablasts, immune repertoire

## Abstract

Lyme disease (*Borrelia burgdorferi* infection) is increasingly recognized as a significant source of morbidity worldwide. Here, we show that blood plasmablasts and CD27^−^ memory B cells are elevated in untreated Lyme disease, with higher plasmablast levels associated with more rapid resolution of clinical symptoms. Stronger serum reactivity to surface proteins and peptides from *B. burgdorferi* was also associated with faster resolution of clinical symptoms. Through molecular identifier-enabled antibody heavy-chain sequencing of bulk B cells and single-cell paired-chain antibody sequencing of blood plasmablasts, we characterized immunoglobulin gene usage patterns specific to *B. burgdorferi* infection. Recombinantly expressed antibodies from expanded lineages bound *B. burgdorferi* antigens, confirming that these clones are driven by the infection. Furthermore, recombinant sequence-derived antibodies were functional, inhibiting growth of *B. burgdorferi in vitro*. Elevations and clonal expansion of blood plasmablasts were associated with rapid return to health, while poor plasmablast responses were associated with a longer duration of symptoms following treatment. Plasmablasts induced by *B. burgdorferi* infection showed preferential antibody gene segment usage, while bulk sequencing of total B cells revealed convergent CDR3 motifs specific to *B. burgdorferi*-infected patients. Our results show that robust plasmablast responses encoding *Bb*-static antibodies are associated with more rapid resolution of Lyme disease, and these antibodies could provide the basis for next-generation therapeutics for Lyme disease.

## Introduction

Lyme disease, caused by *Borrelia burgdorferi* sensu lato (*Bb*) infection, is a debilitating disease in which patients vary in their symptoms, serology, and response to treatment. Although host immunity is not sufficient to clear established untreated disease in many patients, B cells and the antibodies they produce can protect against infection in experimentally infected mice (1, 2). Antibody-mediated protection wanes over time both in mice and humans (3, 4). Nevertheless, our understanding of the role of B cell responses in human Lyme disease remains limited.

In murine models, B cells have been shown to have a role in reducing the duration of active *Bb* infection and the severity of the associated arthritis and carditis (2). Following infection-associated accumulation of CD19^+^ B cells in mouse lymph nodes, there is a disruption in the germinal center architecture, which is accompanied by insufficient anti-*Bb* antibody responses (5, 6). Specifically, experimental infection of mice with *Bb* induces rapid differentiation of B cells into antibody-secreting plasma cells, while long-lived plasma cells and memory B cells are not robustly induced (6–8). Furthermore, the long-term antibody response to influenza vaccination is also diminished in *Bb*-infected, but not *Bb*-immunized mice, demonstrating that this effect extends to exogenous antigens (8). Continual T-dependent production of IgM antibodies, believed to be the result of a non-class switched B-2 cell response, has also been reported in experimentally infected mice (6). Thus, it is believed that *Bb* ensures its own survival in the host by subverting protective B cell responses that would otherwise limit infection. It is unknown whether similar phenomena occur in humans, or how dysregulated human B cell responses may contribute to the heterogeneous disease severity and progression observed among *Bb*-infected patients.

To address the hypothesis that variable B cell responses to *Bb* correlate with variable outcomes following treatment, we characterized B cell populations in *Bb*-infected humans using flow cytometry and antibody repertoire sequencing. We identified robust plasmablast responses encoding *Bb-*static antibodies that are associated with resolution of symptoms following treatment. This work identifies a putative prognostic biomarker associated with return to health following treatment for *Bb* infection and provides insight into an important immune mechanism of *Bb* clearance.

## Materials and Methods

### Study Design

The purpose of this exploratory study was to improve our understanding of the human B cell response to *Bb*, how it changes over time following doxycycline treatment, and how it differs among clinical subsets of patients. We analyzed peripheral blood mononuclear cells (PBMCs) from *Bb*-infected patients over a range of time points spanning the initial (untreated) visit through 2 years following treatment. PBMCs were also collected from healthy controls in the same geographic region (the Mid-Atlantic United States). We assessed the prevalence of B cell populations and performed single-cell plasmablast antibody sequencing as well as unique molecular identifier (UMI) barcode-based bulk heavy-chain sequencing of total B cells. Representative plasmablast antibody sequences were selected for recombinant expression and downstream characterization.

### Inclusion Criteria and Clinical Classification

Human subjects protocols were approved by the institutional review boards of Johns Hopkins University and Stanford University, and all subjects provided written informed consent in accordance with the Declaration of Helsinki. *Bb*-infected patients with an erythema migrans (EM) rash of at least 5 cm and either multiple skin lesions or at least one new-onset concurrent symptom were included in the study. Patients were recruited and enrolled at a suburban clinical practice in Maryland, and those with a previous history of Lyme disease, or preexisting confounding medical conditions associated with fatigue, pain, or neurocognitive symptoms were excluded. Following Infectious Diseases Society of America (IDSA) treatment guidelines, all patients were treated with 3 weeks of oral doxycycline (9). Lyme patients were seen regularly over the course of 2 years for a total of four study visits (at the acute-phase pretreatment visit, at 1-month posttreatment, 6-month posttreatment, and 2-year posttreatment). Samples from healthy controls were collected at an initial visit, 6 months, and 1 year. To differentiate between subjects who returned to health following treatment and those with persistent symptoms, we applied a previously published definition of posttreatment Lyme disease syndrome (PTLDS), which is based on the IDSA’s proposed case definition and incorporates the presence of fatigue, pain, and/or cognitive complaints with functional impact determined by scores on the SF-36, with a composite *T* score of less than 45 (9–11). This definition was applied at all study visits after 6 months from initial diagnosis and treatment. This case definition was chosen on the basis of its previously demonstrated sensitivity for determining the impact of symptoms on the daily function of Lyme disease patients. Subjects with disseminated EM rash were defined as those having more than one visible rash site, while local rash applied to those with a single EM rash site. Re-analysis of published Luminex array data was based on early Lyme disease subjects who were previously described (12).

### Sample Processing and Flow Cytometry

Blood was collected in green-top heparin tubes and processed into PBMCs with Ficoll-Paque PLUS (GE Healthcare, Chicago, IL, USA). PBMC aliquots were frozen in recovery cell culture freezing medium (Thermo Fisher Scientific, Waltham, MA, USA) according to the manufacturer’s instructions and shipped to Stanford for further analysis. After thawing, PBMCs were stained in Hank’s Balanced Salt Solution with 2% fetal bovine serum using the following antibodies: CD20 (clone L27), CD38 (clone HB7), IgD (clone IA6-2), CD3 (clone UCHT1), and CD14 (clone MφP9) from BD Biosciences (San Jose, CA, USA); CD19 (clone HIB19), CD27 (clone O323), and IgM (clone MHM-88) from BioLegend (San Diego, CA, USA); and IgA (clone IS11-8E10) from Miltenyi Biotec (San Diego, CA, USA). Cells were stained for viability by the addition of Sytox blue dye (Thermo Fisher Scientific; Waltham, MA, USA) 10 min before analysis. Single cells were identified by comparing forward scatter area with forward scatter height and gating out cells with increased area relative to height, as compared with the shape plotted by most cells. Plasmablasts were defined as CD19^+/INT^CD3^−^CD14^−^CD20^−^CD27^+^CD38^hi^ live single cells (13). As plasmablasts have a low level of IgG surface expression, IgG-producing plasmablasts were classified by the absence of both IgA and IgM surface staining, and antibody isotypes were further confirmed by gene-specific PCR and antibody constant region sequences. Plasmablasts were single-cell sorted into 96-well plates using a FACSAria II instrument (BD Biosciences).

### Bulk Heavy-Chain Sequencing

Bulk heavy-chain sequencing was performed using a method similar to that described by Turchaninova et al. (14), in which the original 3′ and 5′ of each initial immunoglobulin RNA molecule are each oriented in Read 1 for half the library and in Read 2 for the other half, thus enabling high quality assembly of the full-length VDJ sequence from each initial transcript. Total RNA from Lyme and control PBMCs was extracted with Trizol (Thermo Fisher) and subsequently purified using RNA Clean & Concentrator-5 (Zymo Research) according to the manufacturer’s procedure. Purified RNA was tagged with UMI sequences and a common primer site by template switching during reverse transcription (SmartScribe Reverse Transcriptase, Takara Bio USA, Inc., Mountain View, CA, USA). UMI-barcoded cDNA was amplified with SeqAmp DNA polymerase (Takara Bio USA, Inc.) using the 5′ bridge sequence and gene-specific reverse sequences. This product was then split into two separate reactions per sample, for which Illumina sequencing adapters were added by overlap extension PCR in opposite orientations between the two reactions using Q5 DNA Polymerase (New England Biolabs, Ipswich, MA, USA). Libraries were sequenced at the University of California, Berkeley Genome Sciences Lab (GSL) on a HiSeq 2500 instrument (Illumina, Inc.) for 325 × 275 cycles (rapid run mode). The 325 cycles used for Read 1 is significantly longer than the Illumina-supported read length of 250, and successful runs were highly dependent on the individual instrument. All samples in the study were analyzed on the same specific HiSeq 2500 instrument at GSL. Oligonucleotide sequences used for bulk antibody sequencing are listed in Table S1 in Supplementary Material.

### Bulk Heavy-Chain Sequence Data Analysis

HiSeq 2500 data of total B cell heavy-chain sequences were processed and analyzed using a series of existing tools. Fastq files were produced and de-multiplexed using the Illumina CASAVA/bcl2fastq pipeline. After demultiplexing, fastq files for each sample were concatenated to combine the two lanes, and subsequently processed with MIGEC (15) and MiTools (16) software to combine reads from each umi and build a merged consensus sequence, using pipeline options as described (14). Fastq sequence headers for the UMI-compiled sequences were converted to the pRESTO format (17) before IMmunoGeneTics (IMGT)-HighV-Quest alignment (18) and post-analysis with the Immcantation software suite (Change-O, SHazaM, Alakazam) (19). The rationale for combining MIGEC and Immcantation pipelines was due to the faster performance characteristics of MIGEC in pre-processing large amounts of antibody sequence data from the HiSeq platform, and the flexibility and available functions of the Immcantation post-processing software. Bulk antibody repertoire sequence data are available in GEO (accession number GSE114310).

### Single-Cell Sequencing

After single-cell sorting, plasmablast cDNA was labeled with oligonucleotide barcode sequences, and paired antibody heavy- and light-chain variable regions were sequenced as previously described (20–23). Well-specific barcode sequences (TruGrade DNA oligos; IDT; Coralville, IA, USA) were added to the cDNA of each cell by template switching (Maxima Reverse Transcriptase, Thermo Fisher Scientific). Barcoded cDNA was pooled, gene-specific PCR used to amplify the immunoglobulin heavy- and light-chain variable regions, and MiSeq 2 × 330 paired-end sequencing performed (Illumina, Inc.; San Diego, CA, USA). Oligonucleotide sequences for this method were identical to those reported previously (23).

### Sequence Analysis of Paired-Chain Single-Cell Data

Sequence analysis was performed as previously described (23). Briefly, fastq generation and plate demultiplexing were performed using the onboard MiSeq Generate FASTQ workflow. After quality filtering, paired reads were stitched, separated by well ID, and consensus sequences determined by clustering well ID reads into operational taxonomic units (OTUs) (24). Consensus OTU sequences were analyzed with version 1.3.1 of IMGT HighV-QUEST (18). Clonal families (CF) of plasmablasts were defined based on sharing IMGT-predicted HC (Heavy Chain) and LC (Light Chain) VJ genes and exhibiting >60% amino acid identity within the HC and LC CDR3s. Percent clonality was calculated as the percent of paired sequences that fall into any CF. Single-cell sequence data are available in GEO with accession numbers (*awaiting database acceptance*).

### Recombinant Antibody Expression

Antibodies were selected for recombinant expression based on previously identified criteria including clonal relationships, gene usage, and somatic mutations (20, 23, 25, 26), and were synthesized, cloned, and expressed as previously described (22, 23). To enable comparison of binding using the same secondary antibody, variable domains from all isotypes were expressed on the human IgG1 Fc constant region.

### Characterization of Recombinant Monoclonal Antibody (rmAb) Binding

Plasmablast sequence-derived rmAbs were screened to determine the binding target of each plasmablast antibody using: (i) a custom-developed bead array built with Luminex xMAP technology. This array included 39 peptides and 36 proteins from *Borrelia*, its tick vector (*Ixodes spp*.), and common tick-borne co-infecting pathogens; (ii) two commercial strip-blot assays comprised purified *Borrelia* antigens spotted on a membrane, the Virastripe *Borrelia* Burgdorferi assay and the Virastripe European *Borrelia spp*. assay (Viramed Biotech AG, Planegg, Germany); and (iii) ELISA measurement of rmAb binding to a sonicated native *Bb* strain B31 lysate (Bio-Rad Laboratories, Hercules, CA, USA). Individual rmAbs with strong reactivity on the Luminex array were subsequently validated by ELISA with the recombinant antigen of interest.

### *Borrelia* Growth Inhibition Assay

Low passage *Bb sensu stricto* strain B31 clonal isolate 5A19 was cultured in BSK-II medium at 34°C in an atmosphere of 5% CO_2_, 3% O_2_, and 92% N_2_ as previously described (27). *Bb* spirochetes were grown to early log phase and plated at 5 × 10^6^ cells/mL in 200 µL microtiter plate cultures before treatment with rmAbs (20 μg/well). An influenza hemagglutinin-specific rmAb, derived from sequencing a healthy donor 7 days after Influenza vaccination, was used as the negative control (26). A conventional murine OspA-specific mAb (clone CB10) was used as the positive control. Replicate cultures were collected at time points 0, 24, and 48 h after the initiation of treatment, and growth inhibition was assessed by counting the number of spirochetes per low power field from at least six fields per well.

### Statistical Methods

Pre-processing and analysis of bulk and single-cell antibody sequence data is described in dedicated sections above. All other analyses were performed either with Prism version 7.0c (GraphPad Software, Inc., La Jolla, CA, USA), or with R version 3.3.2 using base/stats functions for statistical analyses and the tidyverse suite (including ggplot2) for data compilation, summary, and visualization. Chord diagrams were drawn with the circlize package in R, and igraphs of cd-hit clustering were produced as previously described (23). Specific statistical tests are detailed in the legend of each figure. One-way ANOVA with Dunnett’s multiple comparisons test was used to determine significance when comparing *Bb*-infected patients at multiple time points with uninfected healthy controls. Comparison of two clinical subsets of *Bb*-infected patients over time was performed using one-way ANOVA with Sidak’s multiple comparisons tests, and Student’s *t*-test was used when comparing a single measurement, such as repertoire stability, between two groups. *P* values of less than 0.05 were considered significant. Analysis of data comparing antibody gene segment usage was performed by Significance Analysis of Microarrays (SAMs) with a Wilcoxon Rank-Sum test, using the samr package in R.

## Results

### Blood Plasmablasts Are Elevated in Lyme Patients Who Return to Health

**Figure 1:**
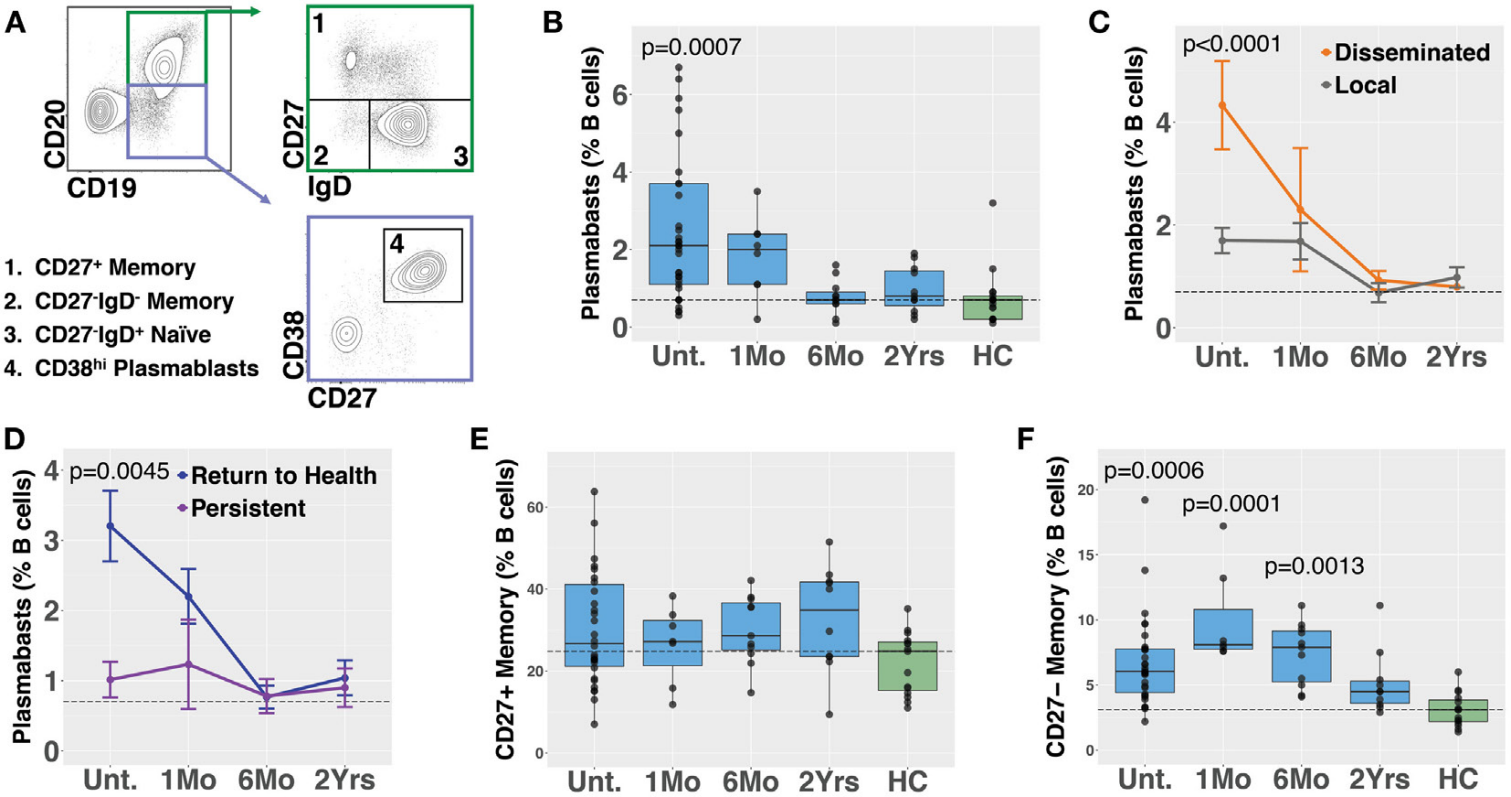
Circulating blood plasmablasts and CD27^−^ memory cells are elevated following *Bb* infection, and plasmablast numbers differ with patient symptoms. Flow cytometry analysis of peripheral blood mononuclear cells was used to analyze B cell populations in *Bb*-infected humans at serial time points starting from the initial (untreated) presentation to 2 years following doxycycline treatment. **(A)** Representative flow cytometry gating of plasmablast and memory B cell populations, starting from CD3^−^CD14^−^ live cells. **(B)** Plasmablast abundance as a percent of CD19^+^ B cells. *P* values shown are versus healthy controls. **(C)** Patients with disseminated erythema migrans rash have significantly higher plasmablast numbers than those with a single rash. **(D)** Patients who reported persistent symptoms exhibited lower plasmablasts levels at the initial visit as compared with those that returned to health. **(E)** Conventional CD27^+^ memory B cells were not significantly altered following infection, but IgD^−^CD27^−^ memory B cells were elevated **(F)**, peaking at 1 month following completion of doxycycline treatment. *P* values were determined using ANOVA with Dunnett’s **(B,F)** or Sidak’s **(C,D)** multiple comparisons tests. Dashed lines represent the median values of healthy controls, and error bars represent the SEs.

Plasmablasts are a population of activated B cells that transiently circulate in the blood during an adaptive immune response, such as to a microbial infection or vaccination [reviewed in Ref. (28)]. We used flow cytometry to analyze plasmablast numbers, as a percent of B cells, in PBMC samples derived from *Bb*-infected (*n* = 32) and healthy control (*n* = 18) subjects (Figures 1A,B). *Bb*-infected subjects were untreated at the initial study visit and then treated for 3 weeks with doxycycline following sample collection at the initial visit; there were no significant differences in age, gender, EM rash, or diagnostic criteria among any of the subject groups (Table 1). Blood plasmablasts, defined by flow cytometry as CD19^+/int^CD3^−^CD14^−^CD20^−^CD27^+^CD38^hi^ live single cells, were significantly elevated in untreated Lyme patients as compared with healthy controls, and returned to baseline levels by 6 months following completion of antibiotic treatment (Figure 1B). Plasmablast numbers differed significantly between defined clinical subsets of *Bb-*infected patients—patients with disseminated EM rash exhibited significantly higher plasmablast numbers compared with those with local EM rash (Figure 1C). Subjects who fully returned to health following doxycycline treatment had significantly higher blood plasmablasts than those who experienced persistent symptoms for at least 6 months following treatment (Figure 1D). To address the potential for variation in B cell numbers to confound plasmablast percentages, we also calculated the number of plasmablasts per 10^3^ live single cells, which confirmed all differences that were observed in plasmablast percentages (Figure S1 in Supplementary Material). There were no significant differences in plasmablast frequency according to age or gender of the subjects (not shown). Together, the data suggest that there is a correlation between initial activation of B cells in response to *Bb-*infection and recovery from the infection after treatment.

**Table 1:** *Bb* infected and healthy control subject characteristics.

	Lyme (total) (*n* = 32)	Lyme (persistent) (*n* = 7)	Lyme (return to health) (*n* = 25)	Healthy (*n* = 18)
Female sex, *n* (%)	17 (53%)	3 (43%)	14 (56%)	10 (56%)
Age, mean ± SD	50.4 ± 15.2	47.9 ± 18.0	51.1 ± 14.7	58.7 ± 11.7
Disseminated rash, *n* (%)	11 (34%)	1 (14%)	10 (40%)	–
Two-tier positive, *n* (%)	26 (81%)	5 (71%)	21 (84%)	–

### Circulating CD27^−^ but Not CD27^+^ Memory B Cells Increase Following *Bb* Infection

Memory B cells are critical to the development of recall responses to infectious disease. They can be divided into subpopulations based on their expression of surface markers including IgD and CD27. In most cases, CD27^+^ switched (IgD^−^) and unswitched (IgD^+^) memory B cells predominate. Nevertheless, there is a population of CD27^−^ memory B cells (differentiated from naïve cells by a lack of surface IgD) that express somatically hypermutated antibody genes (29). *Bb* infection is associated with deficient memory B cell responses in mice (8). In humans, we observed that while CD27^+^ memory B cell numbers did not increase following *Bb* infection (Figure 1E), CD27^−^IgD^−^ memory B cells exhibited significant increases following *Bb* infection, peaking at 1 month following completion of doxycycline treatment (Figure 1F). In contrast to plasmablasts, memory B cell populations did not differ according to resolution of symptoms (Figure S2 in Supplementary Material). To address the potential for variation in B cell numbers to confound memory B cell percentages, we also calculated the number of memory B cells per 10^3^ live single cells, which confirmed all differences that were observed in memory B cell percentages (Figure S1 in Supplementary Material).

### Published Luminex Data Support a Correlation Between Robust B Cell Responses and Return to Health Following Treatment for Lyme Disease

**Figure 2:**
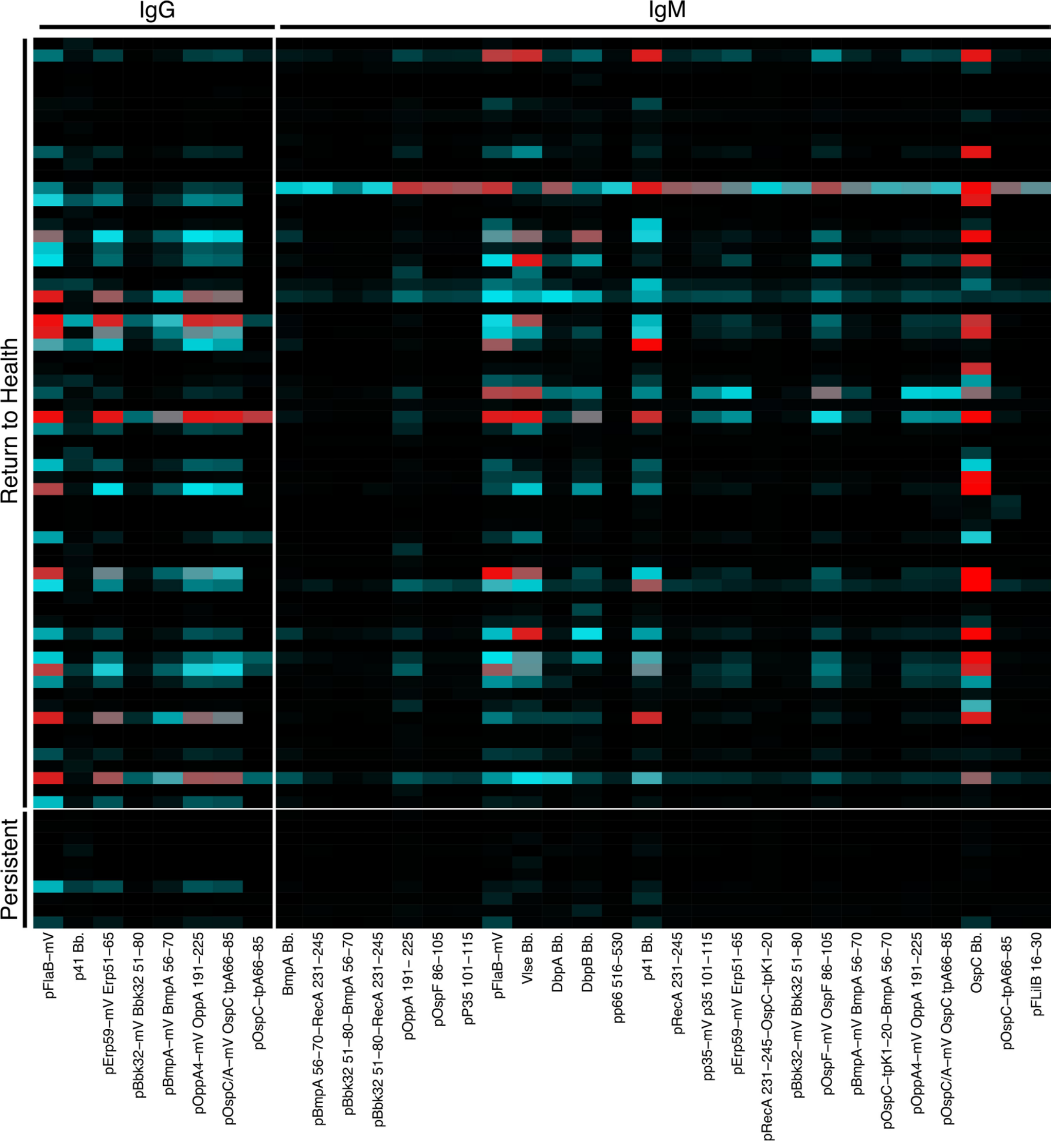
Serum reactivity to *Bb* surface proteins is stronger in patients who return to health following treatment. Serum IgG and IgM reactivity to 62 *Bb* surface proteins and peptides was measured in a previously published study to assess the diagnostic utility of these markers (12). Based on observed differences in B cell populations (Figure 1), serum Luminex data were reanalyzed to assess differences between recently infected Lyme disease patients who reported persistent symptoms and those who returned to health following treatment. Mean fluorescence intensities (MFI) were compared using Significance Analysis of Microarrays, and the MFI of markers with significantly different reactivity are displayed on the heatmap.

We previously reported a bead-based array for “digital” serodiagnosis of Lyme disease, which identifies a high proportion of infected patients using a 10-antigen panel selected from an original set of 62 *Bb* surface proteins and peptides (12). This study focused on the development of a panel appropriate for the diagnosis of Lyme disease in all affected subjects and did not include significant biological analysis of clinical subgroups. Based on the observed differences in B cell activation between groups of Lyme disease patients (Figure 1), we reanalyzed reactivity to all 62 antigens to identify differences in serum reactivity at the initial study visit between recently infected Lyme disease patients who developed persistent symptoms (*n* = 10) and those who promptly returned to health following treatment (*n* = 64, Figure 2). Nineteen subjects overlapped between the Luminex diagnostic data (12) and this study. Patients who returned to health following treatment had significantly higher IgG and IgM reactivity to 8 and 26 markers, respectively (Figure 2).

### Antibody Repertoire Characteristics of Total (Bulk) B Cells Show Increased Clonal Expansion in Patients Who Return to Health

**Figure 3:**
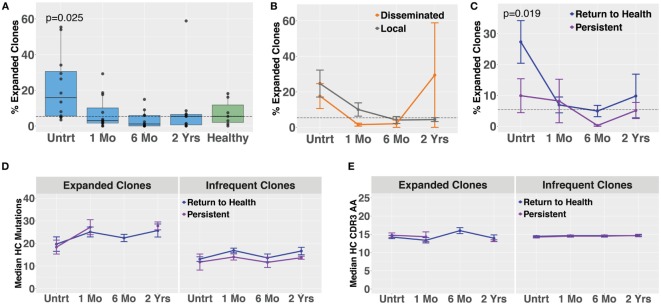
Bulk heavy-chain antibody repertoire sequencing shows increased clonal expansions in untreated *Bb*-infected humans. Total RNA from peripheral blood mononuclear cells was used for unique molecular identifier barcode-based sequencing of immunoglobulin heavy chains, yielding a mean of 49,293 full-length error-corrected VDJ sequences per subject. Percentage of expanded clones was analyzed across serial time points from the initial (untreated) presentation to 2 years following the completion of doxycycline treatment. **(A)** Degree of clonal lineage expansion was significantly increased in untreated Lyme disease. Percent clonality was calculated as the percent of clones that expanded to comprise more than 1% of the sequences at a given time point for each patient. **(B)** Degree of clonal lineage expansion was similar between patients with disseminated and local erythema migrans rash. **(C)** Patients with a shorter duration of symptoms (return to health) had significantly greater clonality as compared with those who experienced persistent symptoms following treatment. **(D)** The median number of somatic hypermutations and **(E)** median CDR3 amino acid lengths were similar between patient groups, for both expanded and infrequent clones. *P* values are from ANOVA with Dunnett’s **(A)** or Sidak’s **(C)** multiple comparisons tests. Dashed lines represent the median value of healthy controls, and error bars represent the SE.

Molecular identifier-based heavy-chain antibody sequencing was performed on mRNA isolated from unsorted PBMCs derived from *Bb-*infected patients and healthy controls (*n* = 12, *n* = 7, respectively). Unique B cell clones were defined as sequences sharing HC V and J gene usage as well as 84% amino acid similarity in the CDR3 region. Clonally expanded families were defined as unique clones making up more than 1% of all sequences derived from a patient at a given time point. The threshold of 84% CDR3 similarity was determined based on the local minimum of a bimodal distribution of nearest neighbor distances, similar to previously described methods used in the Change-O software suite (19). Antibody clonal expansion, measured as the percent of clones that were part of an expanded family, was significantly increased in untreated Lyme disease patients as compared with healthy controls (Figure 3A). There was no difference in clonality between subjects with disseminated rash and those with a single local rash (Figure 3B). However, in samples collected before treatment, subjects who would later return to health had significantly more clonally expanded families than those who experienced persistent symptoms (Figure 3C), paralleling our observation for circulating plasmablast numbers (Figure 1D). There were no significant differences in somatic hypermutation or heavy-chain CDR3 length among any of the groups. However, somatic hypermutation was significantly higher among expanded clones as compared with infrequent clones (<1% of patient-derived sequences at an individual time point) for healthy controls as well as *Bb*-infected patients at all time points (Figures 3D,E). Due to the number of persistent Lyme subjects with no expanded clones at later time points, it was not possible to calculate mutation numbers or CDR3 lengths for expanded clones of this group at the 6-month time point. Together, these data suggest that the degree of clonal expansion seen in the total B cell population may be indicative of ability to recover from *Bb*-infection.

### Plasmablast Repertoire Characteristics Differ Among Subsets of *Bb*-Infected Humans

**Figure 4:**
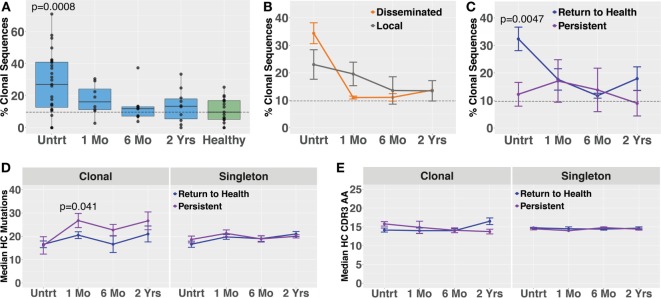
Paired-chain plasmablast repertoire sequence characteristics are similar to those observed in bulk B cell heavy-chain sequencing. Blood plasmablasts were single-cell sorted and tagged with cell-specific barcode oligonucleotides to enable sequencing of their paired heavy- and light-chain genes, yielding a mean of 436 full-length variable region paired sequences per subject. Plasmablasts were defined as CD19^int/+^CD3^−^CD33^−^CD14^−^CD20^int/−^CD27^+^CD38^hi^ live single cells. **(A)** Degree of clonal lineage expansion was significantly increased in plasmablasts from untreated Lyme disease. Percent clonality was calculated, across serial time points, as the percent of cells that were part of an expanded clonal family out of the total number of paired sequences from that time point. **(B)** Clonality showed a trend of increase in plasmablasts from patients with disseminated erythema migrans rash. **(C)** As was seen with bulk B cells, patients with a shorter duration of symptoms (return to health) exhibited significantly greater plasmablast clonality than those with persistent symptoms. **(D)** Mutations from germline and **(E)** CDR3 amino acid lengths showed minor variations among patient subsets. *P* values are ANOVA with Dunnett’s **(A)** or Sidak’s **(B–E)** multiple comparisons tests. Dashed lines represent the median value of healthy controls, and error bars represent SE.

Blood plasmablasts from Lyme disease (*n* = 27) and healthy controls (*n* = 16) were single-cell sorted, and paired-chain antibody repertoire sequencing was performed using an oligonucleotide cell barcoding method we have previously developed and applied to a diverse set of diseases (20–22, 25, 26, 30). For plasmablasts, unique clones were defined by cell barcodes, and clonally expanded families defined as clones sharing HC and LC V and J gene segment usage and 60% CDR3 amino acid similarity. A lower threshold of CDR3 similarity was used for paired HC + LC sequencing because the probability of a clonal relationship is higher when both HC + LC V and J genes match (rather than solely HC) and because comparison of both HC + LC CDR3 sequences provides more information than HC CDR3 alone. The degree of clonal expansion before treatment was significantly higher in plasmablasts derived from *Bb*-infected humans as compared with healthy controls (Figure 4A). Plasmablast antibody clonal expansions also trended higher among subjects with disseminated EM rash as compared with those with a single rash site (Figure 4B). Similar to what was observed in total B cells, plasmablast clonal expansion was significantly higher in untreated subjects who later returned to health as compared with those who experienced persistent symptoms (Figure 4C). Among clonally expanded sequences, patients with persistent symptoms had significantly more mutations from the predicted germline sequence at 1 month following treatment, as compared with patients who returned to health (Figure 4D). There were no differences in somatic hypermutation or heavy-chain CDR3 length between the groups at other time points (Figures 4D,E). Using the alakazam package in R (19), CDR3 amino acid sequences were assessed for eight biochemical properties including amino acid bulk, charge, polarity, and grand average hydropathy (GRAVY). Biochemical properties of the CDR3 amino acid sequences were similar across groups for both bulk B cells and single plasmablasts (Figures S3 and S4 in Supplementary Material). Thus, clonal expansion of both plasmablasts and total B cells is indicative of poor predicted outcomes following treatment for *Bb* infection.

### Plasmablast Repertoires Show Preferred Antibody Gene Segment Usage

**Figure 5:**
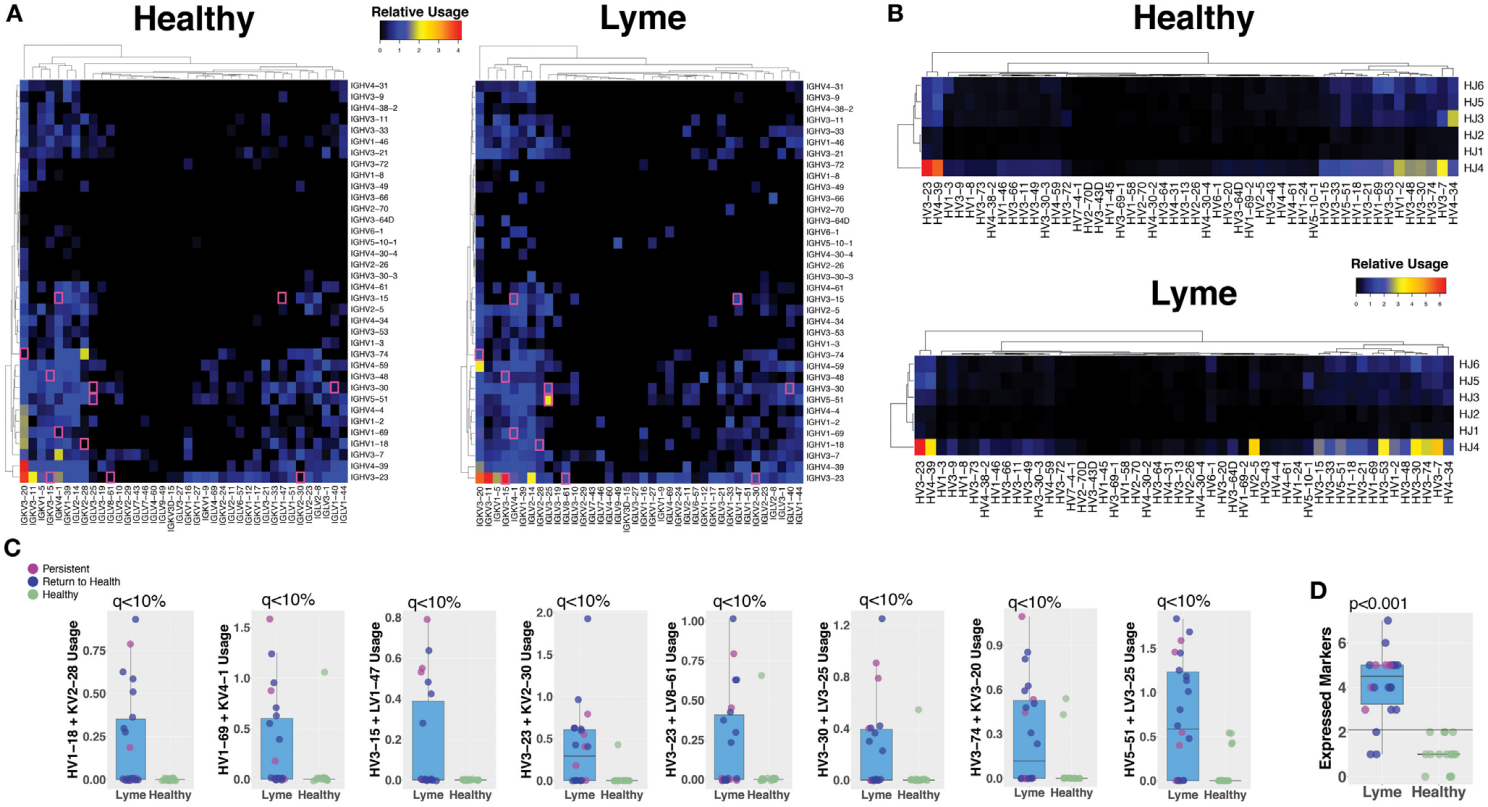
Sequencing the paired-chain plasmablast antibody repertoires, but not HC sequencing of bulk blood B cells, reveals preferred gene segment usage in Lyme disease. Predicted V and J gene segment usage by plasmablasts (heavy and light chain) and total B cells (heavy chain) were identified by alignment with the IMmunoGeneTics database. The percent usage of each gene combination was compared between Lyme disease patients and healthy controls. **(A)** The relative number of paired plasmablast sequences using a given heavy- and light-chain V and J gene combination was compared between untreated Lyme disease patients and healthy controls, and the median value from each group is displayed on the heatmap. The presented heatmap is subsetted to show only the most frequent combinations from among all subjects, with significantly different combinations (*q* < 10%) highlighted in a pink box. **(B)** Heavy-chain gene segment usage for bulk B cell sequencing was calculated as the relative usage of a given predicted heavy-chain V and J gene combination. **(C)** Representative gene combinations preferentially enriched or reduced in Lyme disease were plotted to show individual subject values and statistics. **(D)** Number of expressed V-gene-combination markers for each patient, of eight selected markers. Using a cutoff of >2 markers, the maximum number observed for healthy controls provides 84% sensitivity and 100% specificity for Lyme disease. *Q* values were calculated by Significance Analysis of Microarray with the Wilcoxon Rank-Sum test **(A–C)**. *P* values were calculated using the Student’s *t*-test **(D)**.

Paired-chain antibody sequences from blood plasmablasts and bulk heavy-chain sequences from total B cells were aligned using IMGT HighV-QUEST with the IMGT database, which enables prediction of the germline gene segments that were used for each transcript (18). Preferred usage of specific antibody and TCR gene segments has previously been reported in autoimmune and infectious diseases. Plasmablasts from subjects with untreated *Bb* infection showed differences in preferred gene segment usage when compared with the gene usage observed in healthy controls (Figure 5A). However, gene usage among total B cells was similar between the groups (Figure 5B). SAMs identified significant differences between plasmablast usage of 12 individual gene combinations between Lyme disease patients and healthy controls, while no significant differences were observed between patients with persistent symptoms and those who returned to health (Figure 5C). For example, usage of IGHV3-74 + IGKV3-20 and IGHV5-51 + LV3-25 was observed in sequences from the majority of *Bb*-infected patients, but rarely observed among healthy controls. When assessed in combination, usage of at least three out of eight selected V gene combinations was predictive of Lyme disease with 84% sensitivity and 100% specificity, an improvement upon currently available diagnostic assays (Figure 5D).

### Total B Cells From Lyme Subjects Show Usage of Disease-Specific CDR3 Sequence Motifs

**Figure 6:**
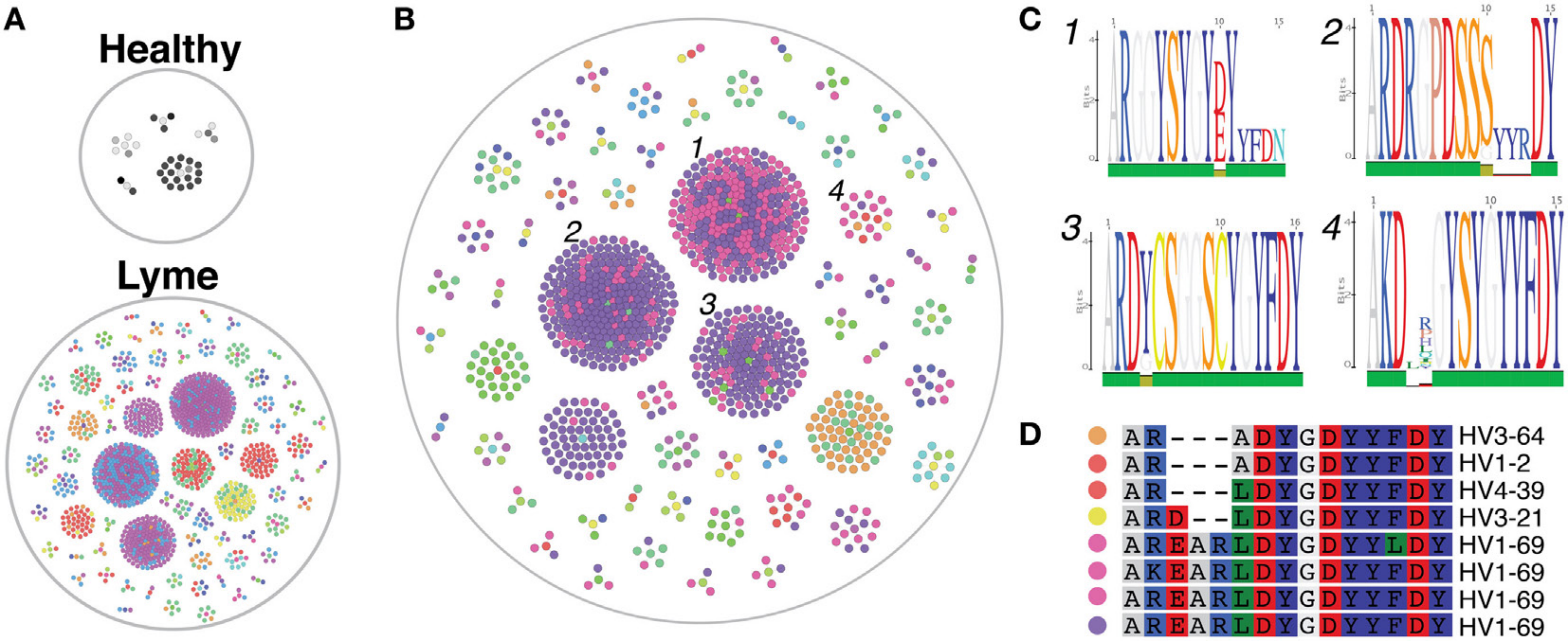
Heavy-chain immunoglobulin sequencing of bulk mRNA from peripheral blood mononuclear cells (PMBCs) derived from untreated *Bb-*infected patients reveals disease-specific CDR3 sequence motifs. Bulk heavy-chain CDR3 sequences from PMBCs of untreated Lyme disease patients and healthy controls were clustered using the cd-hit algorithm to identify convergent sequence motifs. A total of 379,457 full-length error-corrected VDJ sequences were included in the analysis. Each dot represents a sequenced antibody clone, with colors representing individual subjects. **(A)** Clusters were filtered based on those containing at least three separate subjects, and the size and number of these convergent clusters visualized using the igraph package in R. Healthy controls revealed only five cross-patient clusters, while numerous convergent clusters were revealed untreated Lyme disease patients. **(B,C)** Convergent clusters present in at least three separate Lyme subjects **(B)**, but not present in any healthy controls were visualized using igraph, and individual sequences from four clusters with multiple subjects were aligned to display specific sequence motifs **(C)**. **(D)** Sequence alignment of cluster 4, which includes five separate *Bb-*infected patients, including the predicted V gene used by each clone. Colored dots correspond to matching subjects in panel **(B)**.

The cd-hit algorithm (31, 32) was used with a cutoff of 0.8 to identify clusters of heavy-chain CDR3 amino acid sequences with motifs shared by at least three subjects among either healthy controls or Lyme patients (Figure 6A). Only five small clusters were shared among three or more healthy controls, while >60 shared clusters of varying size were observed among at least three individuals with Lyme disease. Of these Lyme clusters, several were observed in one or more healthy controls, while the majority was unique to *Bb*-infected patients (Figure 6B). Cross-patient clusters were aligned to enable visualization of their sequence motifs (Figure 6C). In many cross-patient clusters, motifs arose from usage of different V genes (Figure 6D), suggesting that convergent evolution of the antibody response toward binding a common antigen underlies this observation.

### A Subset of Clones Persist in the Circulation of Both Lyme Disease and Healthy Controls

To investigate the stability of antibody repertoires over time following *Bb* infection, we compared individual clonal expansions across time points spanning the initial (untreated) visit through 2 years following antibiotic treatment. Persistent plasmablast clones were visualized using chord diagrams (Figure S5 in Supplementary Material), and differences were quantified by calculating the percent of clones from the initial visit that persisted after 6 months. Neither plasmablast nor bulk PBMC B cell repertoires exhibited significant differences in repertoire stability between the groups (Figure S5 in Supplementary Material).

### Plasmablast Clones Encode *Bb*-Specific Antibodies

**Figure 7:**
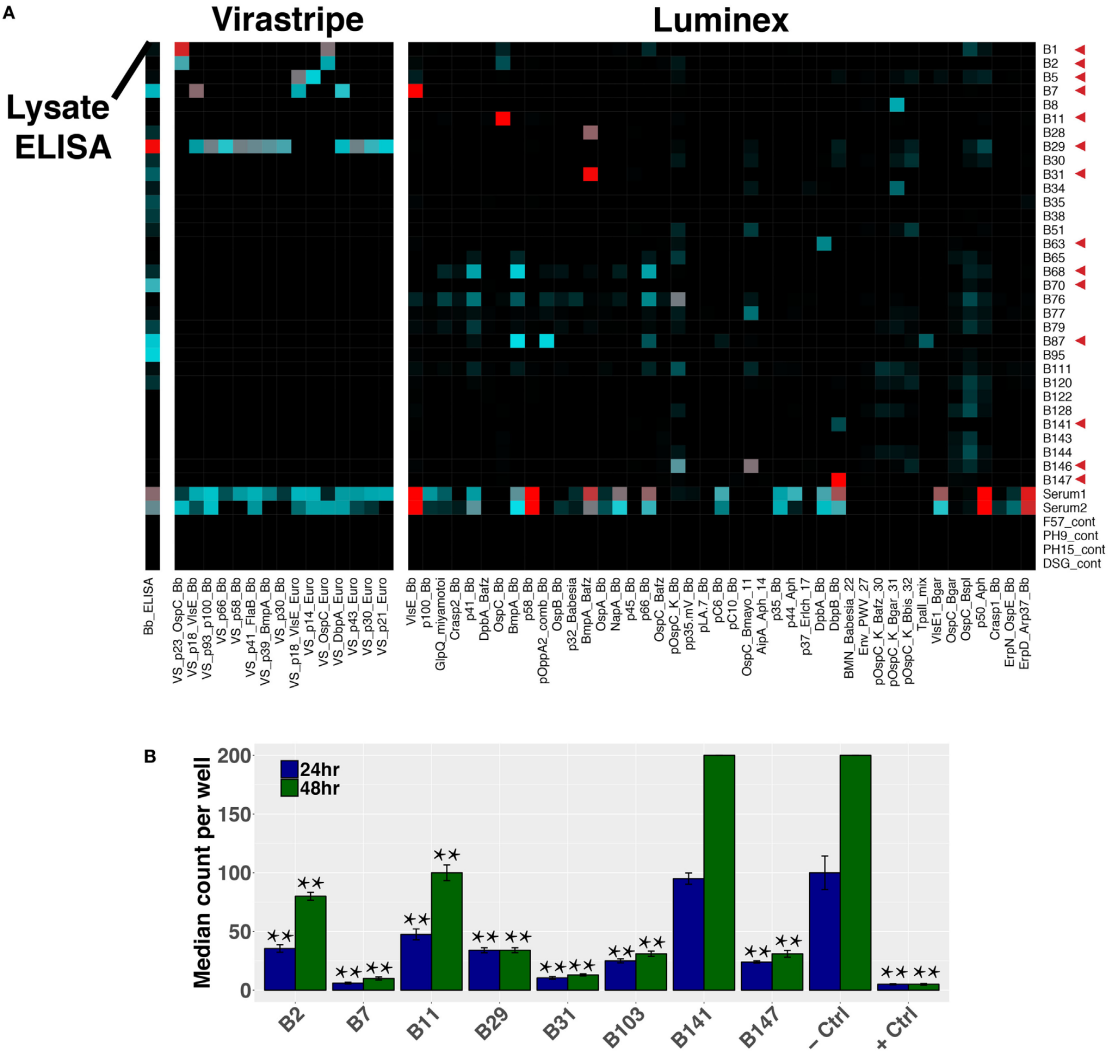
Plasmablast-derived recombinant monoclonal antibodies (rmAbs) from both acute and persistent B cell clones bind *Bb*-specific antigens and inhibit growth *in vitro*. Sequence-derived antibodies representative of the identified clonal families were synthesized, cloned, and recombinantly expressed. Recombinant expression enabled characterization of the rmAb binding targets with an in-house ELISA and multiplex bead-based assay, a commercial strip-blot assay, and in *in vitro Bb* growth assays. **(A)** Custom Luminex assay containing whole proteins and peptides derived from *Bb* and related species, sonicated *Bb* B31 lysate ELISA, and Virastripe strip-blot assay. Red arrows indicate successful ELISA validation of rmAb binding. **(B)** rmAbs were added to *Bb* cultures to assess their ability to inhibit *Bb* growth *in vitro*. The negative control was an rmAb specific for Influenza hemagglutinin, and the positive control was a conventional murine mAb specific for *Bb* OspA (clone CB10). *P* values were determined by one-way ANOVA with Tukey’s *post hoc* test relative to negative control, **P < 0.01.

Paired-chain antibody repertoire sequencing of individual plasmablasts enables expression and characterization of sequence-derived rmAbs. Of the available paired sequences from all subjects, 144 native HC–LC sequence pairs were selected for expression. Selection was based on sequence characteristics including clonal relationships, presence at multiple time points or at the initial (untreated) time point, isotype, mutations from the predicted germline sequence, and CDR3 length. Selected sequences were gene synthesized and cloned into a mammalian expression vector containing the human IgG1 constant region, followed by expression by transient transfection. To enable equal binding and functional comparisons of the rmAbs, all rmAbs were expressed on the human IgG1 Fc regardless of the native isotype. Purified rmAbs were analyzed to identify binding specificity using a sonicated lysate ELISA, a commercial strip-blot assay, and a custom magnetic bead-based assay containing 75 different proteins and peptides (Figure 7A). Subsets of *Bb-*infected patient rmAbs from single time points and from persistent families bound to *Borrelia* antigens including VlsE, DbpA, DbpB, OspC, and others (Figure 7A).

### Sequence-Derived rmAbs Inhibit *Bb* Growth *In Vitro*

Sequence-derived rmAbs that bound *Borrelia* surface antigens were further characterized to assess their ability to inhibit *Bb* growth after 24 and 48 h (Figure 7B). Of the rmAbs assayed, seven mediated statistically significant inhibition of *Bb* growth *in vitro* at both 24 and 48 h. The antigen targets of *Bb*-static rmAbs are listed in Table S2 in Supplementary Material.

## Discussion

The mechanisms by which B cells control *Bb* infection in humans are poorly understood. Here, we describe a key role for plasmablasts that express *Bb*-static antibodies and report B cell characteristics associated with more rapid resolution of symptoms following doxycycline treatment in *Bb*-infected humans. These observations provide new insights into immune mechanisms that correlate with successful antibiotic-mediated clearance of *Bb* and return to health.

Plasmablasts are activated B cells generated by germinal center responses and are released into the blood where they transiently circulate before homing to secondary lymphoid and diseased tissues. The release of plasmablasts into the blood combined with their transient nature provides a window into the B cells generated in an acute ongoing immune response, differentiating them from the >10^8^ total immunoglobulin specificities in a human’s B cell repertoire (33). We herein identify plasmablasts as a key B cell population that correlates with resolution of *Bb* infection and Lyme disease in humans. Although many aspects of B cell responses to *Bb* are deficient in experimentally infected mice, the presence of affinity maturation and/or CDR3 convergence in a subset of cells has not specifically been addressed. Thus, our findings are consistent with and extend the findings in experimentally infected mice, in which antibody-secreting cells are rapidly induced following infection and the presence of B cells is necessary for the resolution of arthritis and carditis (2, 6).

We utilized both single-cell paired chain and bulk heavy-chain antibody repertoire sequencing to investigate *Bb*-induced B cell responses. Increased clonal expansion at the initial study visit, before treatment, was observed in both bulk and single-cell datasets. However, many *Bb*-associated differences, including preferred germline gene segment usage and persistently expanded clones, were only detectable in plasmablasts, highlighting the importance of characterizing this population. Furthermore, paired-chain antibody repertoire sequencing enables expression of representative rmAbs followed by downstream binding and functional characterization of the encoded sequences. We identified 12 immunoglobulin HC V + LC V gene combinations that were significantly overrepresented in the plasmablasts of *Bb*-infected patients, including IGHV3-74 + IGKV3-20 and IGHV5-51 + LV3-25. In contrast to the preferred gene usage combinations observed in paired-chain sequencing data, no significantly preferred gene usage combinations were identified in single-chain data from either total PBMC B cells or sorted plasmablasts.

Applying a targeted panel of eight preferred HC V + LC V gene combinations gave 84% sensitivity and 100% specificity for *Bb* infection, a significant improvement over the current CDC-recommended diagnostic regimen. More than three million Lyme disease tests are performed in the United States each year, at an estimated cost of $492 million dollars (34). These tests fail to capture more than 50% of patients during the earliest stages of infection, although their sensitivity is significantly improved 30 days following manifestation of clinical symptoms (35). Prompt diagnosis is particularly important for Lyme disease, as delays in treatment are associated with an increased likelihood of persistent symptoms following treatment (36). It will be important to systematically assess the presence of these V gene combinations in large validation cohort to further assess their diagnostic utility.

In addition to plasmablasts, CD27^−^ memory B cell responses have been associated with infection and autoimmunity (37–41). We show herein that these cells expand following *Bb* infection in humans. The CD27^−^ memory B cell population is not well understood, but it has been reported to increase in autoimmune diseases including rheumatoid arthritis, systemic lupus erythematosus, and multiple sclerosis, as well as during infections with rotavirus or human immunodeficiency virus (37–41). Evidence supporting the identity of this population as memory B cells includes studies showing that these cells are physically larger than antigen-inexperienced CD27^−^IgD^+^ cells, as well as the observation of class-switching and somatic hypermutation in this population (29, 42). Further investigation is needed to characterize the encoded antibodies and functional properties of CD27^−^ memory B cells following *Bb* infection.

Limitations of this study include the limited number of patients sequenced. Comparisons of *Bb*-infected versus healthy individuals and persistent versus resolving symptoms included a sufficient number of subjects to identify statistically significant differences. However, comparisons of subjects with disseminated versus local EM rash may have been limited by the small proportion of patients with a disseminated rash. Given the identified differences between these groups, future studies will be needed to further characterize patients with disseminated EM rash. While this study included directed sequencing of plasmablasts, as well as total PBMC B cell sequencing, we did not perform directed sequencing of CD27^−^ memory B cells, which we observed to increase following infection. Further characterization of this population will also fall under the scope of a future study.

Following the recent use of monoclonal antibody therapies in Ebola and respiratory syncytial virus infections, anti-microbial monoclonal antibodies are re-emerging as an important therapeutic strategy for emerging infectious diseases (43, 44). While vaccine and infection-induced antibody responses have the potential to protect against and clear *Bb* infection, in many cases such responses are inadequately induced or maintained. We show here that in patients who lack a robust B cell response, doxycycline treatment alone does not ensure full recovery. Passive treatment or prophylaxis with *Bb*-specific monoclonal antibodies could promote clearance of *Bb*, facilitate doxycycline-mediated pathogen clearance, reduce the likelihood of developing PTLDS, and/or prevent infection in highly endemic areas such as the Northeastern United States. Improvements in our understanding of the factors affecting the pharmacokinetics of therapeutic antibodies have increased the feasibility of prophylactic use, as mAb half-lives can be extended beyond 1 month (45, 46), potentially enabling seasonal protection from a single dose. Although cost may be a limiting factor in the usefulness of *Bb-*specific therapeutic mAbs, future improvements in mAb manufacturing techniques and development of *Bb-*specific mAbs with extremely high affinities (thus requiring a low dose) may mitigate this limitation.

The presence and underlying pathobiology of persistent Lyme disease symptoms has historically proven controversial. However, a significant body of work from multiple research groups now supports the verity of this syndrome, demonstrates that live spirochetes persist under certain conditions following antibiotic treatment of rhesus macaques (47), and identifies CCL19 and IL-23 as biomarkers associated with the failure of symptom resolution (48, 49). This study adds blood plasmablast levels, B cell clonal expansion, and serum reactivity to a subset of *Bb* surface proteins to the set of cellular characteristics that are associated with development of symptoms that can arise following *Bb* infection.

Herein, we demonstrate that following *Bb* infection elevations and clonal expansion of blood plasmablasts are correlated with rapid return to health, while poor plasmablast responses are associated with a longer duration of symptoms following doxycycline treatment. *Bb*-induced plasmablasts exhibited preferential antibody gene segment usage, while bulk sequencing of total B cells revealed convergent Lyme-specific CDR3 motifs. Resolution of symptoms following antibiotic treatment was associated with robust plasmablast responses encoding *Bb-*static antibodies, suggesting that plasmablast-derived anti-*Bb* antibodies could provide the basis for next-generation antibody-based therapeutics for Lyme disease.

## Data Availability Statement

The antibody repertoire sequence data reported in this paper have been deposited in GEO (accession number GSE114310). All other data will be made available by the authors, without undue reservation, to any qualified researcher.

## Ethics Statement

Human subjects protocols were approved by the institutional review boards of Johns Hopkins University and Stanford University, and all subjects provided written informed consent in accordance with the Declaration of Helsinki.

## Author Contributions

Conceptualization: LB, WR, JNA, and MS; methodology: LB, JZA, and SE; software: LB and SE; formal analysis: LB; investigation: LB, JZA, DM, and RC; resources, ME, JNA, MS, and WR; data curation: LB, JZA, and AR; writing—original draft preparation: LB and WR; writing—reviewing and editing: JZA, DM, AR, SE, RC, ME, JNA, MS, and WR; visualization: LB and WR; supervision: LB, WR, ME, JNA, MS, and WR.

## Conflict of Interest Statement

WR owns equity in, is a consultant to, and is a member of the Board of Directors of Atreca, Inc. All other authors declare no competing interests.
