# Supplementary material for: Robust B Cell Responses Predict Rapid Resolution of Lyme Disease
Source: Front Immunol. 2018 Jul 18;9:1634. doi: 10.3389/fimmu.2018.01634 (PMC6060717; doi:10.3389/fimmu.2018.01634)
Supplement: Supplementary file 1 [file data_sheet_1.PDF]

Supplementary Material

Robust B cell responses predict rapid resolution of Lyme disease

Lisa K. Blum[1,2], Julia Z. Adamska[1,2], Dale S. Martin[3], Alison W. Rebman[4], Serra E. Elliott[1,2], Richard R.L. Cao[1], Monica E. Embers[3], John N. Aucott[4], Mark J. Soloski[4], William H. Robinson[1,2]*

[1]Stanford University School of Medicine, Stanford, CA, USA
[2]VA Palo Alto Healthcare System, Palo Alto, CA, USA
[3]Division of Bacteriology and Parasitology, Tulane National Primate Research Center, Tulane University Health Sciences Center, Covington, LA, USA
[4]Lyme Disease Research Center, Division of Rheumatology, Department of Medicine, Johns Hopkins University School of Medicine, Baltimore, MD, USA

*** Correspondence:**
William H. Robinson
w.robinson@stanford.edu

Supplementary Figures

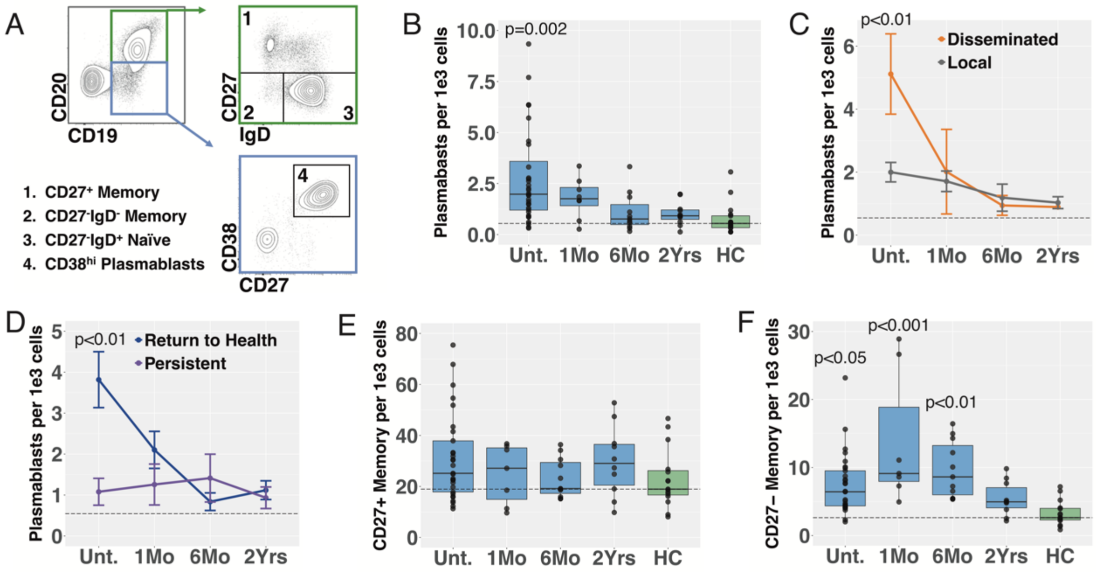

Supplementary Figure 1. **Absolute numbers of circulating blood plasmablasts and CD27^{-} memory cells are elevated following *Bb* infection, and plasmablast numbers differ with patient symptoms.** Flow cytometry analysis of PBMCs was used to analyze B cell populations in *Bb*-infected humans at serial time points starting from the initial (untreated) presentation to two years following doxycycline treatment. Cell population numbers were calculated as the number per 10^3 live single cells. (A) Representative flow cytometry gating of plasmablast and memory B cell populations, starting from CD3^{-}CD14^{-} live cells. (B) Plasmablast numbers per 10^3 live single cells. *P* values shown are versus healthy controls. (C) Patients with disseminated EM rash have significantly higher plasmablast numbers than those with a single rash. (D) Patients who reported persistent symptoms exhibited lower plasmablasts numbers at the initial visit as compared to those who returned to health. (E) Conventional CD27^{+} memory B cells were not significantly altered following infection, but IgD^{-}CD27^{-} memory B cells were elevated (F), peaking at one month following completion of doxycycline treatment. *P* values were determined using ANOVA with Dunnett's (B, F) or Sidak's (C, D) multiple comparisons tests. Dashed lines represent the median values of healthy controls, and error bars represent the standard errors. Dashed lines represent the median value of healthy controls, and error bars represent standard error. Statistical comparison was performed by ANOVA.

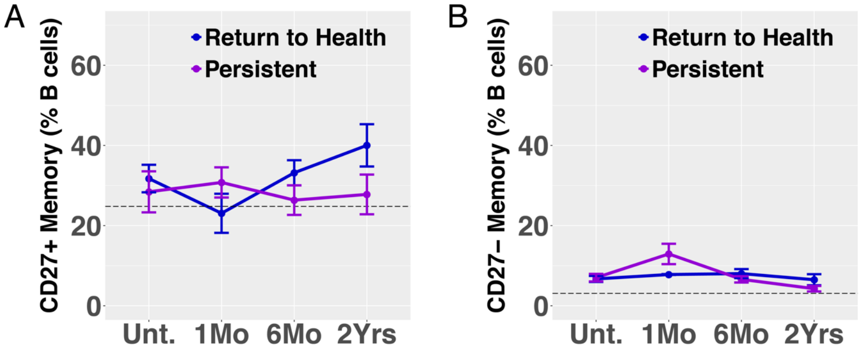

Supplementary Figure 2. Memory B cell populations do not differ according to patient symptoms. Neither (A) $CD27^+$ memory B cells nor (B) $CD27^-$ memory B cells differ between *Bb*-infected patients who report persistent symptoms and those who return to health following doxycycline treatment. Dashed lines represent the median value of healthy controls, and error bars represent standard error. Statistical comparison was performed by ANOVA.

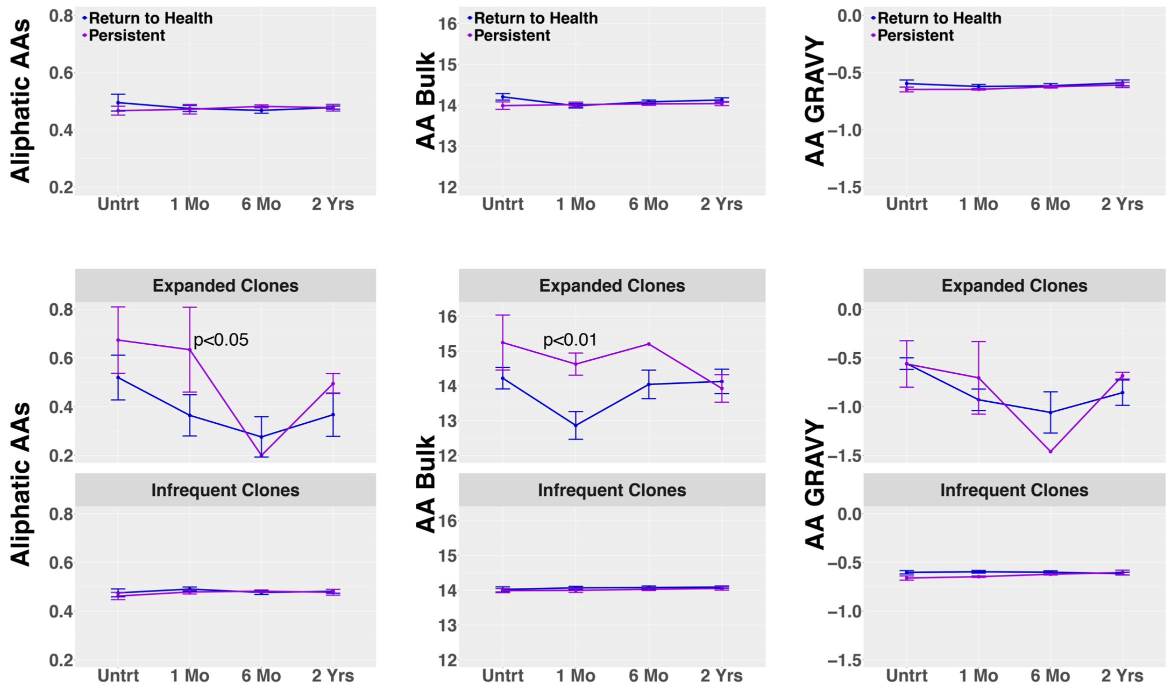

Supplementary Figure 3. CDR3 amino acid properties in bulk B cell sequencing differ between expanded and infrequent clones. The total proportion of aliphatic amino acids, amino acid bulk, and grand average hydropathy (GRAVY) in the heavy-chain CDR3s of *Bb*-infected patients did not differ according to the resolution of symptoms. However, for both groups of *Bb*-infected patients, expanded clones trended towards having CDR3s that are aliphatic and have greater amino acid bulk than the CDR3 sequences of infrequent clones. P values were determined by ANOVA with Sidak's multiple comparisons test, and error bars represent standard error.

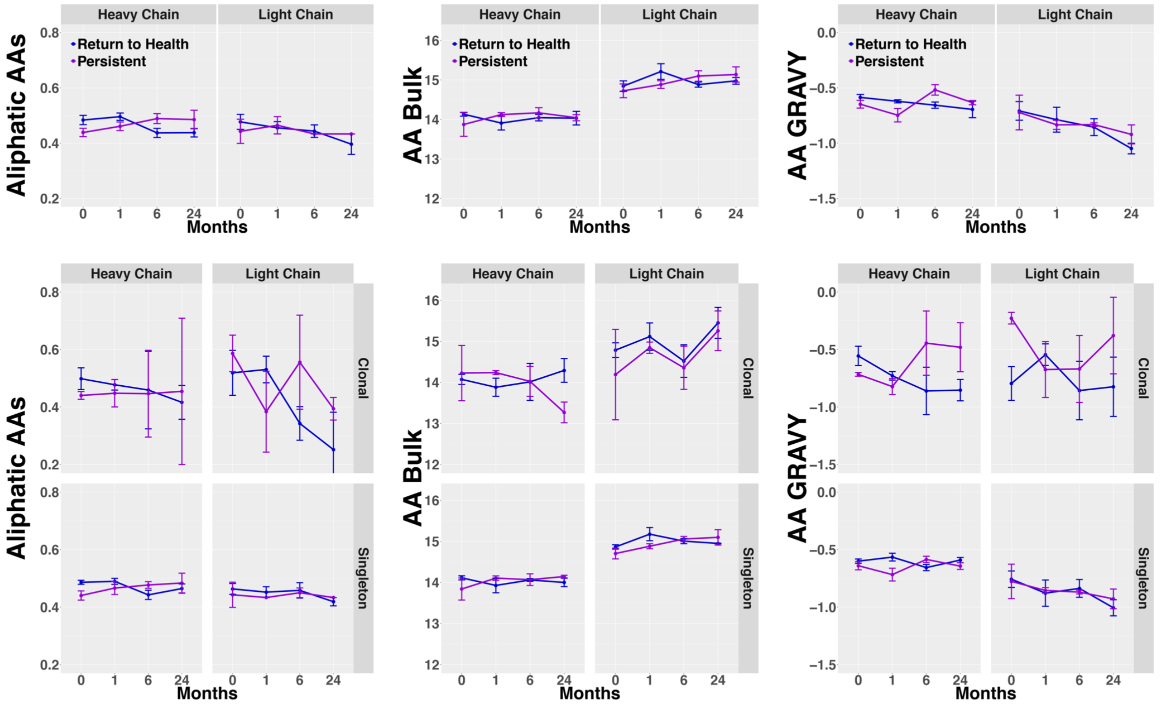

Supplementary Figure 4. CDR3 amino acid properties in paired-chain plasmablast sequencing datasets are similar between Bb-infected patients and healthy individuals. The proportion of aliphatic amino acids, amino acid bulk, and grand average hydropathy (GRAVY) were not significantly different between patients with persistent symptoms and those who returned to health. Statistical comparison was performed by ANOVA, and error bars represent standard error.

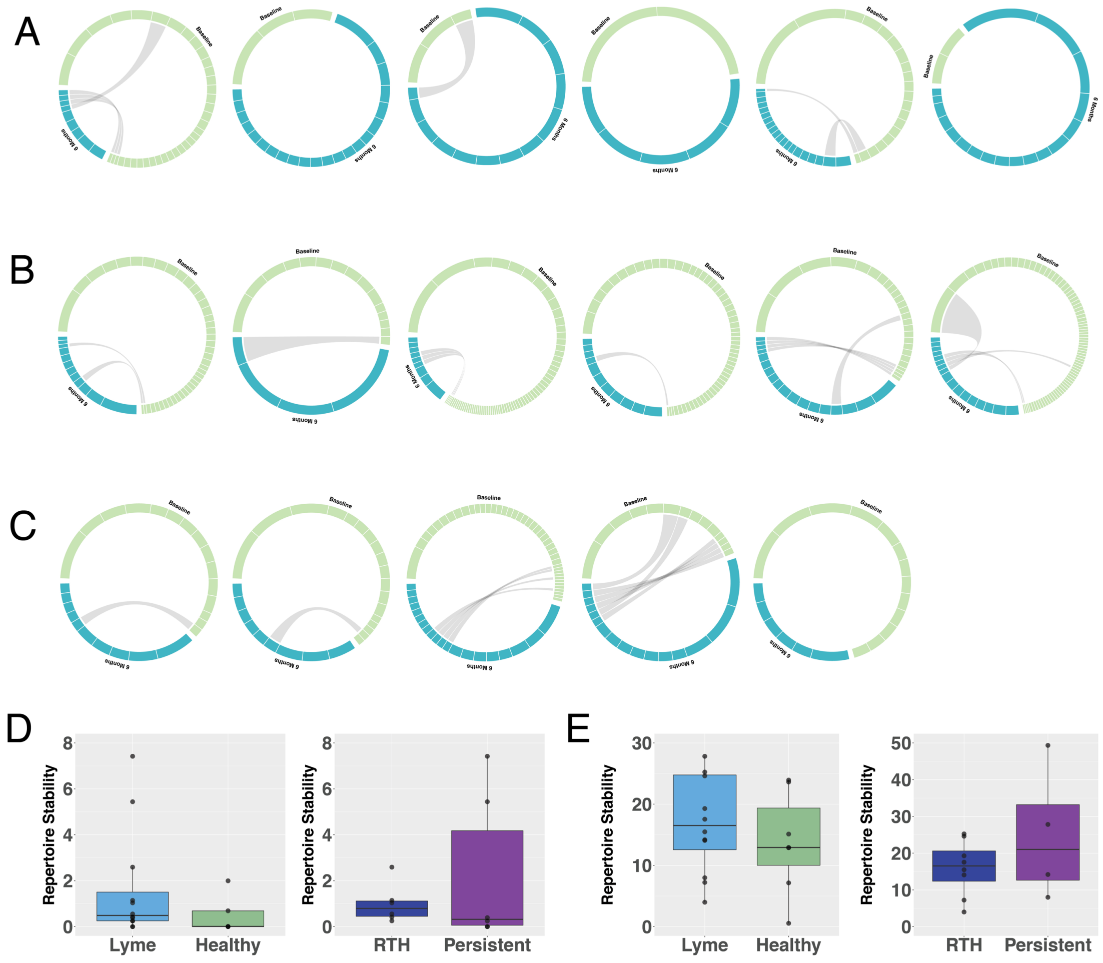

Supplementary Figure 5. Plasmablast clonal expansions persist in both *Bb*-infected humans and healthy controls. Bulk heavy-chain and single-cell plasmablast sequencing was compared across two time points (baseline and 6 months) for both *Bb*-infected patients and healthy controls. Clonal families were defined based on shared V and J gene usage and percent CDR3 amino acid similarity (84% for heavy chain sequencing and 60% for paired-chain sequencing). (A) Healthy control, (B) Lyme-Persistent, and (C) Lyme-Return to Health chord diagrams of plasmablast repertoires in which each outer segment represents one clonally expanded family at each time point. Chords connect clonal lineages shared across-time points. (D-E) Stability was measured as the percent of clonal families (D, plasmablasts) or expanded clones (E, bulk B cells in PBMCs) that persist across both time points. No statistically significant differences were observed, as determined using the Student's t-test.

Supplementary Table 1. Oligonucleotide sequences used in the study.

Name	Sequence	Step
M1mod_dT	/5SpC3/GGAGTCGTGTCACGTACAGAGTCATCTTTTTTTTTTTTTTTTTTTTTTTTTTTTTTVN	RT
RT_RACE1	/5Me-isodC/AAGCAGTGGTATCAACGCAGAGNNNNNNNNNNDVVHCTTGrGrGrG/3SpC3/	RT
RT_RACE2	/5Me-isodC/AAGCAGTGGTATCAACGCAGAGNNNNNNNNNNCTTGrNrGrG/3SpC3/	RT
Bridge_Fwd	AAGCAGTGGTATCAACGCAGAG	PCR1
M1_Rev	/5SpC3/GGAGTCGTGTCACGTACAGAGTCATC	PCR1
G_GSP1	TCTTGTCCACCTTGGTGTTGCTG	PCR2 (both mixes)
A_GSP1	ATTCGTGTAGTGCTTCACGTGG	PCR2 (both mixes)
M_GSP1	CTCTCAGGACTGATGGGAAGC	PCR2 (both mixes)
PCR2A_R1	/5SpC3/TCGTCGGCAGCGTCAGATGTGTATAAGAGACAGNNNAAGCAGTGGTATCAACGCAGAG	PCR2 (mix A)
PCR2B_R2	/5SpC3/GTCTCGTGGGCTCGGAGATGTGTATAAGAGACAGNNNAAGCAGTGGTATCAACGCAGAG	PCR2 (mix B)
P5_short	AATGATACGGCGACCACCGAGATC	PCR3 (mix A)
PCR3A_R1	/5SpC3/ATGATACGGCGACCACCGAGATCTACAC-**[I5 index]**-TCGTCGGCAGCGTCAGATGTGTATAAG	PCR3 (mix A)
PCR3A_GSP_IgG	CAAGCAGAAGACGGCATACGAGAT-**[I7 index]**-GTCTCGTGGGCTCGGAGATGTGTATAAGAGACAGnnnnGTAGTCCTTGACCAGGCAGCC	PCR3 (mix A)
PCR3A_GSP_IgG_short	CAAGCAGAAGACGGCATACGAGAT-**[I7 index]**-GTCTCGTGGGCTCGGAGATGTGTATAAGAGACAGnnnnAAGACSGATGGGCCCTTGGTG	PCR3 (mix A)
PCR3A_GSP_IgA	CAAGCAGAAGACGGCATACGAGAT-**[I7 index]**-GTCTCGTGGGCTCGGAGATGTGTATAAGAGACAGnnnnAGAAGCCCTGGACCAGGCA	PCR3 (mix A)
PCR3A_GSP_IgA_short	CAAGCAGAAGACGGCATACGAGAT-**[I7 index]**-GTCTCGTGGGCTCGGAGATGTGTATAAGAGACAGnnnnAGAAGACCTTGGGGCTGGTCG	PCR3 (mix A)
PCR3A_GSP_IgM	CAAGCAGAAGACGGCATACGAGAT-**[I7 index]**-GTCTCGTGGGCTCGGAGATGTGTATAAGAGACAGnnnnAAGTCCTGTGCGAGGCAGC	PCR3 (mix A)
PCR3A_GSP_IgM_short	CAAGCAGAAGACGGCATACGAGAT-**[I7 index]**-GTCTCGTGGGCTCGGAGATGTGTATAAGAGACAGnnnnGAGACGAGGGGGAAAAGGGTT	PCR3 (mix A)
P7_short	CAAGCAGAAGACGGCATACGAGAT	PCR3 (mix B)
PCR3B_R2	/5SpC3/CAAGCAGAAGACGGCATACGAGAT-**[I7 index]**-GTCTCGTGGGCTCGGAGATGTGTATAAG	PCR3 (mix B)
PCR3B_GSP_IgG	ATGATACGGCGACCACCGAGATCTACAC-**[I5 index]**-TCGTCGGCAGCGTCAGATGTGTATAAGAGACAGnnnnGTAGTCCTTGACCAGGCAGCC	PCR3 (mix B)
PCR3B_GSP_IgG_short	ATGATACGGCGACCACCGAGATCTACAC-**[I5 index]**-TCGTCGGCAGCGTCAGATGTGTATAAGAGACAGnnnnAAGACSGATGGGCCCTTGGTG	PCR3 (mix B)
PCR3B_GSP_IgA	ATGATACGGCGACCACCGAGATCTACAC-**[I5 index]**-TCGTCGGCAGCGTCAGATGTGTATAAGAGACAGnnnnAGAAGCCCTGGACCAGGCA	PCR3 (mix B)
PCR3B_GSP_IgA_short	ATGATACGGCGACCACCGAGATCTACAC-**[I5 index]**-TCGTCGGCAGCGTCAGATGTGTATAAGAGACAGnnnnGAAGACCTTGGGGCTGGTCG	PCR3 (mix B)
PCR3B_GSP_IgM	ATGATACGGCGACCACCGAGATCTACAC-**[I5 index]**-TCGTCGGCAGCGTCAGATGTGTATAAGAGACAGnnnnAAGTCCTGTGCGAGGCAGC	PCR3 (mix B)
PCR3B_GSP_IgM_short	ATGATACGGCGACCACCGAGATCTACAC-**[I5 index]**-TCGTCGGCAGCGTCAGATGTGTATAAGAGACAGnnnnGAGACGAGGGGGAAAAGGGTT	PCR3 (mix B)

Supplementary Table 2. Binding targets and commercial antigen sources of Bb-static rmAbs.

Human rmAb	Antigen	Antigen Source
B2	*B. burgdorferi* OspC	Surmodics, Inc., Eden Prairie, MN, USA
B7	*B. burgdorferi* VlsE	Surmodics, Inc., Eden Prairie, MN, USA
B11	*B. burgdorferi* OspC	Surmodics, Inc., Eden Prairie, MN, USA
B29	*B. burgdorferi* sonicate lysate	Bio-Rad, Inc., Hercules, CA, USA
B31	*B. burgdorferi* BmpA	Surmodics, Inc., Eden Prairie, MN, USA
B103	*B. burgdorferi* NapA	Surmodics, Inc., Eden Prairie, MN, USA
B147	*B. burgdorferi* sonicate lysate	Bio-Rad, Inc., Hercules, CA, USA
